# Data treatment methods for real-time colorimetric loop-mediated isothermal amplification reactions

**DOI:** 10.1038/s41598-023-40737-x

**Published:** 2023-09-01

**Authors:** Edson Yu Sin Kim, Louise Matiê Imamura, Bruna Winkert Raddatz, Santiago Pedro Timm Soares, Victor Henrique Alves Ribeiro, Diego Rinaldi Pavesi Nicollete, Erika Bergamo Santiago, Marcus Vinícius Mazega Figueredo, Bernardo Montesanti Machado de Almeida, Sergio Renato Rogal

**Affiliations:** Hilab, Rua José Altair Possebom, 800-CIC, Curitiba, Paraná 81270-185 Brazil

**Keywords:** Data processing, Laboratory techniques and procedures

## Abstract

With the SARS-CoV-2 pandemic and the need for affordable and rapid mass testing, colorimetric isothermal amplification reactions such as Loop-Mediated Isothermal Amplification (LAMP) are quickly rising in importance. The technique generates data that is similar to quantitative Polymerase Chain Reaction (qPCR), but instead of an endpoint color visualization, it is possible to construct a signal over a time curve. As the number of works using time-course analysis of isothermal reactions increases, there is a need to analyze data and standardize their related treatments quantitatively. Here, we take a step forward toward this goal by evaluating different available data treatments (curve models) for amplification curves, which allows for a cycle threshold-like parameter extraction. In this study, we uncover evidence of a double sigmoid equation as the most adequate model to describe amplification data from our remote diagnostics system and discuss possibilities for similar setups. We also demonstrate the use of multimodal Gompertz regression models. Thus, this work provides advances toward standardized and unbiased data reporting of Reverse Transcription (RT) LAMP reactions, which may facilitate and quicken assay interpretation, potentially enabling the application of machine learning techniques for further optimization and classification.

## Introduction

Isothermal amplification strategies are revolutionizing diagnostics, especially in Point-of-Care settings, as they are simpler to perform, are generally more resistant to contaminants in the sample, and may work without sample purification steps. They also require less instrumentation in general^1^. Loop-Mediated Isothermal Amplification (LAMP)^2^ and more specifically the reverse transcriptase (RT) containing RT-LAMP variation has been applied in Point-of-Care and Testing (POCT)^3–6^ scenarios due to its great sensitivity and fast reaction time.

LAMP consists of a technique for nucleic acid amplification by a strand-displacement DNA polymerase with two to three pairs of primers designed to create loops in the synthesized single-strand DNA, and consequently new amplification initiation sites. Due to the high amount of DNA synthesis in the LAMP reaction—approximately 11 μg of DNA in a 25 μL reaction, 55 times the yield of PCR (Polymerase Chain Reaction) technique^7^—it is possible to visually differentiate a positive response.

The high DNA amount coupled with colorimetric pH dyes has enabled naked-eye detection of nucleic acid amplification in RT-LAMP reaction tubes^6,8–10^. As deoxynucleotides (dNTPs) are incorporated in the synthesized DNA strand, protons are released as a by-product, causing a decrease in the pH of the reaction and a color change of the dye. This enabled mass testing with PCR-like sensitivity and specificity during the start of the COVID-19 pandemic. The only requirement for such a diagnostic kit is keeping the reaction temperature constant, with the use of a water bath or a thermal block. Such tests, however, may introduce ambiguity and subjectivity to the analysis, due to weak reactions resulting in hard-to-interpret intermediate colors. The subjectivity of naked-eye visualization for other colorimetric methods has already been reported, leading to an invalid rate of 25%^11^.

RT-LAMP reactions do not happen on cycles and instead happen continuously. Real-time data acquisition, with either colorimetric or fluorescent dyes, yields Michaelis–Menten-like graphs similar to qPCR (quantitative PCR) amplification plots, which consist of cycles against normalized fluorescence^12^⁠. Therefore, it should be possible to analyze RT-LAMP reaction kinetics using the same established models used in qPCR, and to extract parameters that relate to the initial number of target molecules from the curve shape, using similar methods.

In general, the sigmoidal curve profile^13^ resulting from kinetic monitoring of amplification reactions is subdivided into a lag phase—where the product synthesis is still too low to cross the detection threshold—and an exponential phase where the product amount increases rapidly, resulting in an exponential curve profile. Linear and plateau phases follow the exponential phase, due to reaction products and inhibitors accumulating and reagents being consumed. The reaction rate then slows down until there is no more product formation. These phases are usually directly extractable from models such as Richard’s Generalized logistic function^14^, where parameters directly translate to curve geometry (e.g. the term multiplying the time variable is associated with the slope between the baseline and the plateau).

Quantitative parameters from reaction curves may present themselves as relevant information even for qualitative diagnostic assays, as they enable optimization and reagent fine-tuning^15^, enabling primer set performance evaluation and enhanced quality control specifications. These parameters may also be used for pre-classification before analysis by an operator, shortening the turnaround time, a key metric in pandemic-like situations^16,17^. Mathematical modeling of reaction product formation has been used to better discern false positive reactions, although the results relied on endpoint macromolecule population analysis, requiring DNA gel electrophoresis for confirmation^18^.

Time to positivity (TTP) is commonly used as an analog of the CT (cycle threshold) parameter from qPCR assays. This method, however, is subjective and time-consuming, often requiring manual baseline and threshold line adjustment, which may distort results if proper data treatment is not used^19^. Initial amplification-independent effects are sometimes greater than the actual amplification signals in colorimetric reactions, which complicates the use of a threshold. Therefore, the lack of baseline subtraction represents a further difficulty for the direct usage of raw signal data on RT-LAMP reactions.

To compare curves amongst different reaction preparations and locations, a way to standardize and quantify the final curve shape is needed. Here, we perform a systematic evaluation of different regression models—on an in-house data set of more than 400 remote colorimetric RT-LAMP reactions—and determine the best-suited combination for our setup.

## Methods

### Colorimetric RT-lamp reactions

Amplification reactions were prepared using the WarmStart Colorimetric LAMP 2× Mastermix (M1800—New England Biolabs) containing phenol red—a colorimetric pH indicator that enables differentiation of a negative reaction (pink) from a positive reaction (yellow). Primers specific for genes E and N from SARS-CoV-2 (Table S1) were used in the previously reported concentrations^20^⁠, along with 40 mM guanidine hydrochloride (G3272—Sigma Aldrich), in a final reaction volume of 12.5 μL. Controls consisted of pUC57 plasmids containing the E and N sequences from the SARS-CoV-2 genome (Table S2). Positive control reactions contained 1E6 copies of each gene sequence. Reactions were performed using the POCT equipment, Hilab Molecular, at 65 °C for 30 min. The data were collected as a real-time color series and at the end of the procedure two images were generated (Fig. S1). First, an image with the gradual color change of each tube from the beginning to the end of the reaction. And second, a graph with the time in seconds on the X-axis and the color delta of the green channel of the reaction on the Y-axis^15^.

### Amplification datasets

**Figure 1:**
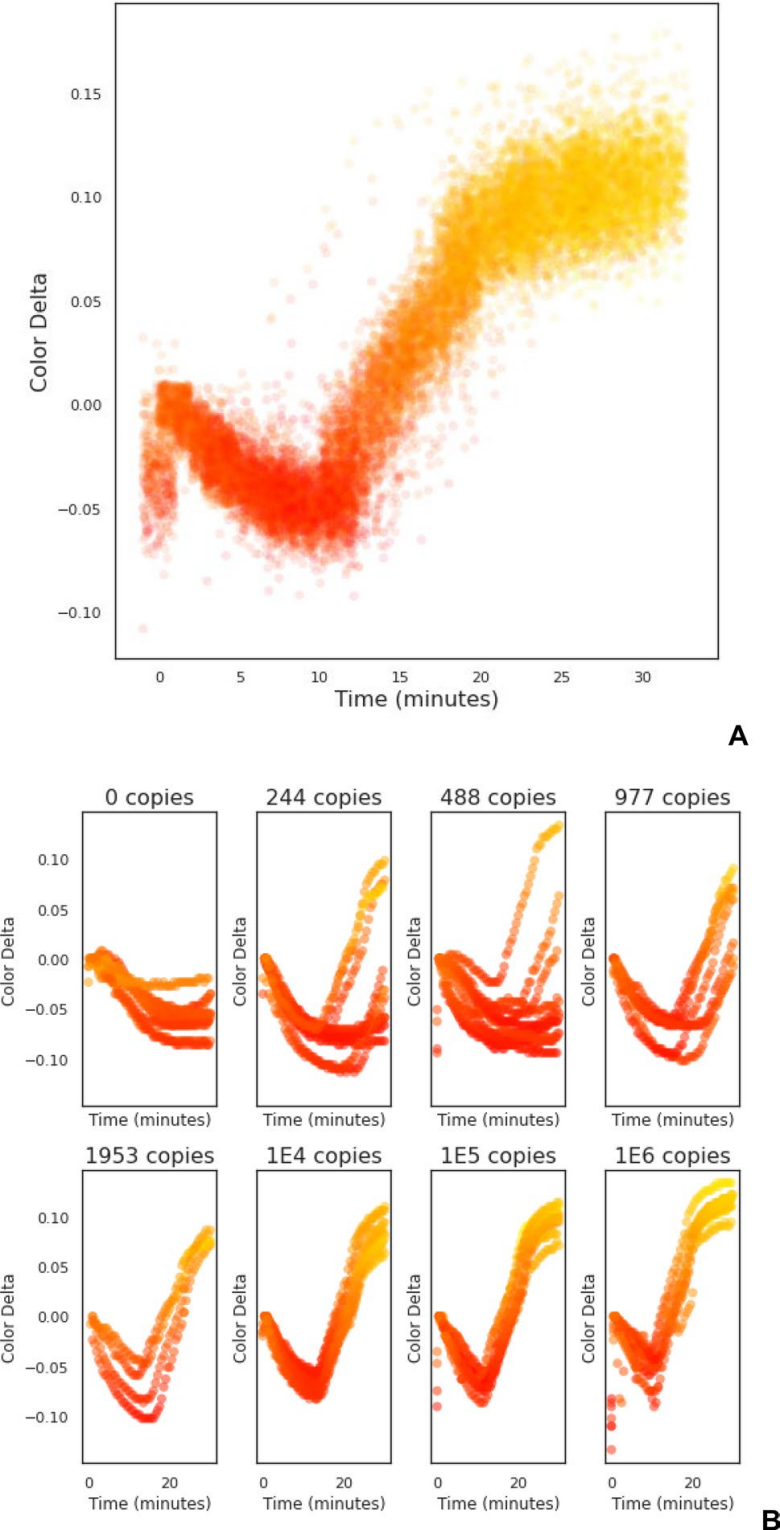
Datasets used to evaluate treatment combinations. Amplification curves are represented as scatter plots with dot colors corresponding to reported RGB values (with adjusted luminance and saturation for better visualization). Y-axis represents the color delta on the green channel. (**A**) Positive control dataset with 482 curves—raw data is available as supplementary material. (**B**) Serial dilution Dataset, initiating from upper left with 0, 244, 488, 977, 1953, 1E4, 1E5, and 1E6 copies of positive control per reaction. Each dilution is at 10 technical replicates.

Raw colorimetric data was obtained as RGB value time series, using the Hilab Molecular device^15^, which is converted to hexadecimal values and sent over remotely from the sample processing location to a central hub responsible for data processing and diagnostic report. Data was reprocessed into RGB values, and the green channel was used to monitor the color change from pink to yellow from a positive reaction. Two datasets of amplification data were used (Fig. 1).

The first dataset consists of roughly 482 positive control amplification curves (Fig. 1A). Positive controls consist of two pUC57 plasmids containing fragments from the SARS-CoV-2 genome, as previously described. This dataset will be referred to as the Positive Control Dataset. A second smaller dataset, with 80 curves, comprised RT-LAMP reactions containing serially diluted controls, from zero to 1E6 copies per reaction (Fig. 1B). Due to the sensitivity of the test, its variability and standard deviation are high at target copy numbers close to the Limit of Detection (LoD)^21,22^ To avoid these non-linear regions, the coefficient of determination (R^2^) was calculated using the logarithm of reactions containing 1E2 to 1E6 copies.

### Colorimetric data processing

All data treatment and visualization was performed using Python. Regression was done using the NumPy^23^, SciPy^24^, sklearn^25^, statsmodels^26^, and pandas^27^ libraries⁠, and graphs were generated using the seaborn library^28^.

Sigmoidal models were combined with a baseline expression (Eq. 1), as described previously^29^⁠. These involved parametrization of curves by non-linear regression using either the least squares method or differential evolution^30^.

Four (Eq. 2) and five (Eq. 3) parameters of logistic regression models were evaluated. Within these models, different coefficients were chosen to represent the measurement of target molecules. For the four-parameter logistic function (Eq. 2), the inflection point and a threshold method were used. For the five-parameter logistic function (Eq. 3), the threshold method was also used alongside the Cy0 method^31^⁠—a method based on the kinetics parameters of the amplification curve. The C_bend_^29^ (the point of maximum curvature) was defined as the maximum of the second derivative of each function being evaluated. A double sigmoid model was constructed by combining two five-parameter curve equations together with the baseline expression.

1 $$B(x) = a.(1-{e}^{-bx}) + c$$

2 $$A(x) = Nmax.\frac{1}{1+{e}^{-r({x}_{t}-x)}} +\mathrm{ c}$$

3 $$A(x) = Nmax.{(\frac{1}{1+{e}^{-r({x}_{t}-x)}})}^{f} +\mathrm{ c}$$

A multimodal Gompertz model (Eq. 4) was also tested, and bounds were set as described previously^32^. Whereas Richard’s models—commonly used to model qPCR curves—require asymmetry parameters added to describe more complex shapes, Gompertz models can do that while maintaining four parameters (accounting for the intercept)^33^. Briefly, a preliminary weighted (weight = w[x], Eq. 5) polynomial regression of time against the double log of k over m_i_ is performed, where k is the last value in the time series (a proxy saturation value) and m_i_ is the raw data series with ‘i’ elements (Eq. 6). This weighting is only applied when obtaining the first guess for the actual final fit, and is implemented to avoid boundary instability when using higher order polynomial regressions. No weighting was used in the objective function when fitting actual candidate models. Coefficients resulting from this preliminary regression are fed as an initial guess for the N coefficients of an (N − 1)-order polynomial that is the exponent Q_β_(t) of the multimodal Gompertz curve, and ɑ which is the natural exponential of the intercept. The polynomial regression was weighted to prioritize the central part of the curve, close to the inflection point and away from the extremities of the available data, where the polynomial fit is unstable.

4 $$A(x)=k.{exp(-\alpha {e}^{-{Q}_{\beta }(t)}})$$

5 $$w\left(x\right)=\left(\frac{0.5 -x^{2}+30x}{225}\right)^{2}$$

6 $$ln \,\, ln \frac{k}{{f}_{\Theta}(t)}= ln \, {\rm a} - {Q}_{\beta }(t)$$

### Statistical evaluation of curve models

A total of 50 curve models were initially evaluated for performance on real-time colorimetric amplification data. First, a dataset composed of 482 different positive control curves (supplementary file)—obtained from remotely performed RT-LAMP reactions containing 1E6 copies of SARS-CoV-2 genome equivalents—was processed using all combinations of curve models. We have also performed colorimetric RT-LAMP reactions using the Hilab Molecular system and plasmid DNA controls in increasing concentrations, similar to primer efficiency curve studies from qPCR methods. It was expected that plotting the log of target copy numbers against relevant parameters (Ct, Cy0) would yield a linear relationship. The coefficient of determination (R^2^) was used as an indicator of data treatment adequacy. The first screening of data treatments excluded statistical outliers from the dilution dataset, while in the second round with the multimodal Gompertz and the double sigmoid, no outliers were removed. Outliers were removed in the first round to improve convergence when using least squares methodologies. The Time To Positivity (TTP) parameter for the double sigmoid was called using the Cy0 method, while the TTP for the Gompertz model was called using the second derivative method.

Akaike Information Criteria (AIC), Bayesian Information Criterion (BIC), and AIC weights (AICw) were calculated and used for comparison^34^. The distribution of called parameters (that is the parameters extracted from the fitted curve, that are correlated to the initial copy number, e.g. Cq and Cy0), was analyzed using Kernel Density Plots (KDE) with the ‘cut’ parameter set to zero—the KDE plot was created using Seaborn. It uses a Gaussian Kernel to represent distributions in a smooth curve. Residual analysis was performed by calculating the Root Squared Error (RSE) between the raw data and the regression curve and by plotting the result for 10 points around the Cy0 point [from the five-parameter regression (Eq. 3)] and the inflection point of the four-parameter regression (Eq. 2), in a logarithmic scale Y-axis.

## Results

### Alternatives to classic sigmoidal models

In a preliminary investigation, using common qPCR models, we evaluated the mean squared error using both least squares and differential evolution regressions, for different logistic models, filters and smoothers. Different regression models yield different patterns of residue distribution, as each performs differently in adjustment. Treatments that best describe the curve shapes should thus exhibit the lowest values of the mean square error of fit. Figure S2 shows the mean of the sum of residuals for different treatment combinations. Overall, differential evolution yields the lowest values of residuals. The four-parameter sigmoid model treatments resulted in the worst fits, while log-logistic models performed better regardless of regression strategy. FIR low-pass seems to negatively affect mean residue values for every scenario. Cubic splines also performed worse than filter-less curves for treatments using differential evolution regression. Only moving average and Butterworth low-pass treatments showed improvement. Differential evolution was used from this point on, as it was shown to be more effective at finding a global minimum. Also, no filters were used from this point on, as they are generally only useful when a high amount of noise or outliers are present in the C_bend_ region, which has been previously demonstrated for qPCR curves^35^. These results are presented in more detail in the supplementary files.

Isothermal amplification commonly exhibits a double sigmoid curve profile, even with reactions that have a single target. This possibly happens because of secondary feedback loops which are present in some kinds of isothermal reactions^36^, though none have been reported for LAMP reactions specifically. This is visible in both fluorescent and colorimetric reporter chemistries when the target nucleic acid initial concentration is high enough (about 3 orders of magnitude higher than the Limit of Detection). Traditional sigmoidal models used for qPCR do not take this into account, giving rise to a systematic fitting error in the exponential bend region.

Two approaches were taken to mitigate this lack of fit. First, a multimodal Gompertz regression model was used, allowing the number of inflection points in the curve to vary. This is accomplished by inserting a higher degree polynomial Q_β_(t) in a traditional Gompertz curve, which produces multiple inflection points as a function of the roots of Q_β_(t). Model selection for this kind of multimodal Gompertz equation involves the fitting of increasingly higher-order polynomials coupled with statistical parameters for model selection. This approach is useful considering that different concentrations of the initial target DNA may generate different numbers of inflection points. Secondly, a double sigmoidal model, with baseline subtraction (Eq. 1), was also evaluated. These models were compared to the four-parameter (Eq. 2) and five-parameter models (Eq. 3), and regression was once again performed with the Positive Control Dataset.

The calling strategy (that is, the way used to calculate a value that is related to copy number, like Cy0 or Cq) for the double sigmoidal model was the same used for the five-parameter models. For the multimodal Gompertz models, the second derivative method was used. A Kernel Density Estimate plot (KDE) was constructed from the mean normalized parameter value for each model, using parameters called when analyzing the Positive Control Dataset. Figure S3 shows the KDE for 14 tested models separated into three groups, based on models and their performances. Two groups are based on multimodal Gompertz, with one exhibiting a mostly monotonic response, and the second with more than one density peak. A third grouping consisted of the classic Richards’s and five-parameter logistic functions (Eq. 3), in addition to the double sigmoid model. The highest density achieved was for the 9th-degree Gompertz model. The middle orders (5th to 8th degree) exhibited erratic behavior and showed multiple density peaks in the KDE plot of called parameters. Four (4p) and five-parameter (5p) (Eqs. 2 and 3, respectively) models performed similarly, with the Double five-parameter (5p) model exhibiting slightly higher density.

**Figure 2:**
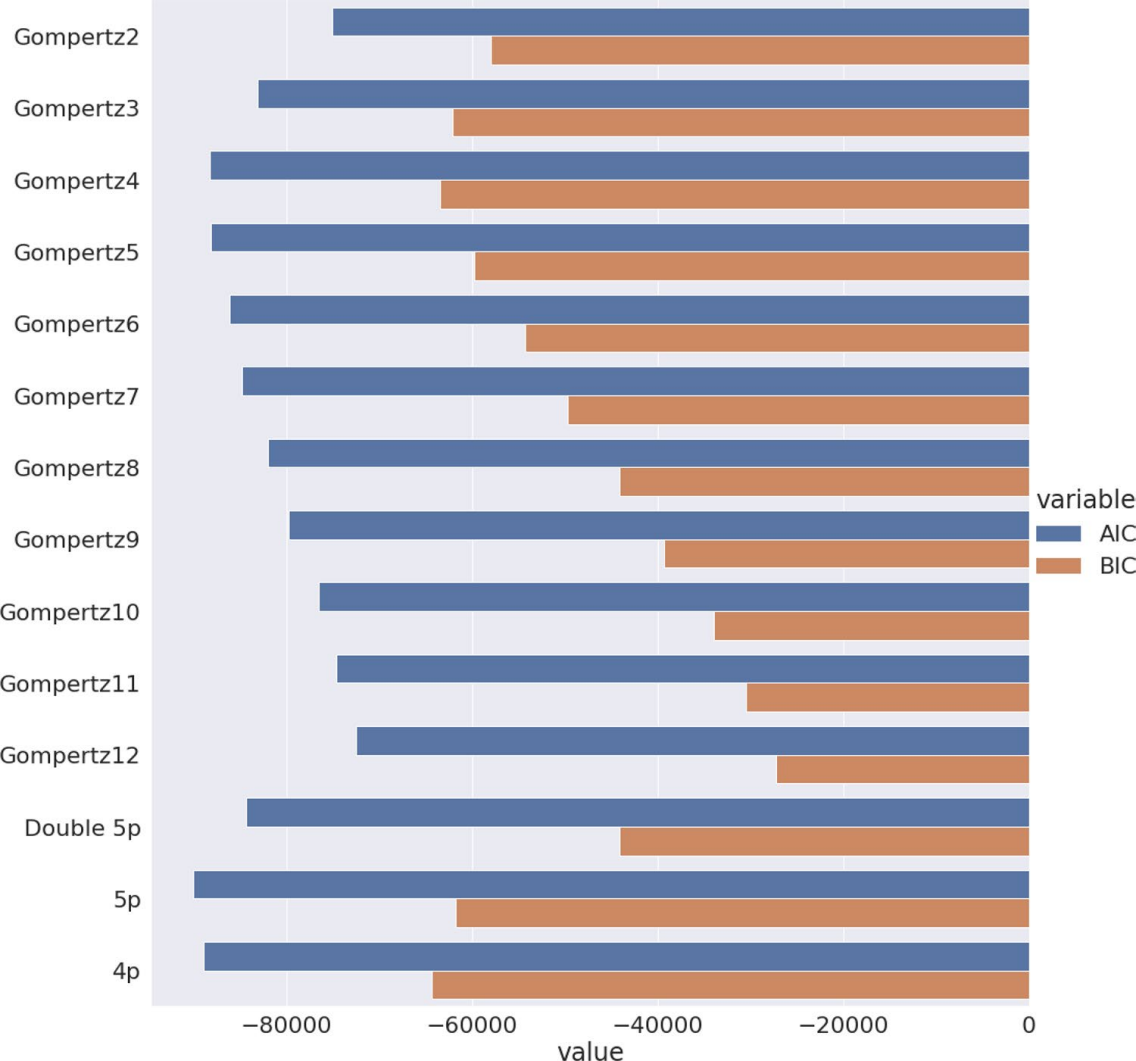
Classic models are generally more efficient concerning the number of parameters. The AIC and BIC increase up to 4 or 5 order polynomial Gompertz models and decline from there on. Richard’s curve variations perform similarly, with the five-parameter (5p) being the best score for AIC and the four-parameter (4p) model the best score for BIC. Vertically are the different curve models tested and horizontally is the value of AIC (in blue) and BIC (in brown) to each model.

Regression models will generally exhibit a greater fit the more parameters it contains, as the number of degrees of freedom is higher. However, this may unnecessarily increase the computational burden and thus increase the calculation time. To account for this fact, Akaike Information Criterion (AIC) and Bayesian Information Criterion^37^ (BIC) may be used, as they balance a model fit score (as the root of the squared sum) against the number of parameters present in the model. A general AIC and BIC were calculated for the entire Positive Control Dataset (Fig. 2). The best-performing model by the AIC was the five-parameter logistic regression (Eq. 3; 5p) followed closely by the 4 and 5-degree polynomial Gompertz model (Gompertz4 and Gompertz5). The BIC has a greater tendency to favor lower numbers of parameters compared to the AIC. In the present analysis, the four-parameter logistic model exhibits a lower score, followed by 5p and Gompertz4.

Another possible way for comparison is the use of Akaike weights (AICw)^34^. For each curve in the Positive Control Dataset, the Akaike weights for all models summed are equal to 1 by definition, which translates AIC scores to a probability of a given model being better, compared to the other models. Normalized KDE Distribution plots of AICw values for the Positive Control Dataset are shown in Fig. S4. Among the best performing (Fig. S4B) are the 4p and 5p, as well as the double-sigmoid with five parameters (Double 5p) models. In general, 4th-degree multimodal Gompertz (Gompertz4) is most often the best performing out of all models tested.

### Analysis of the models using the serial dilutions dataset

**Figure 3:**
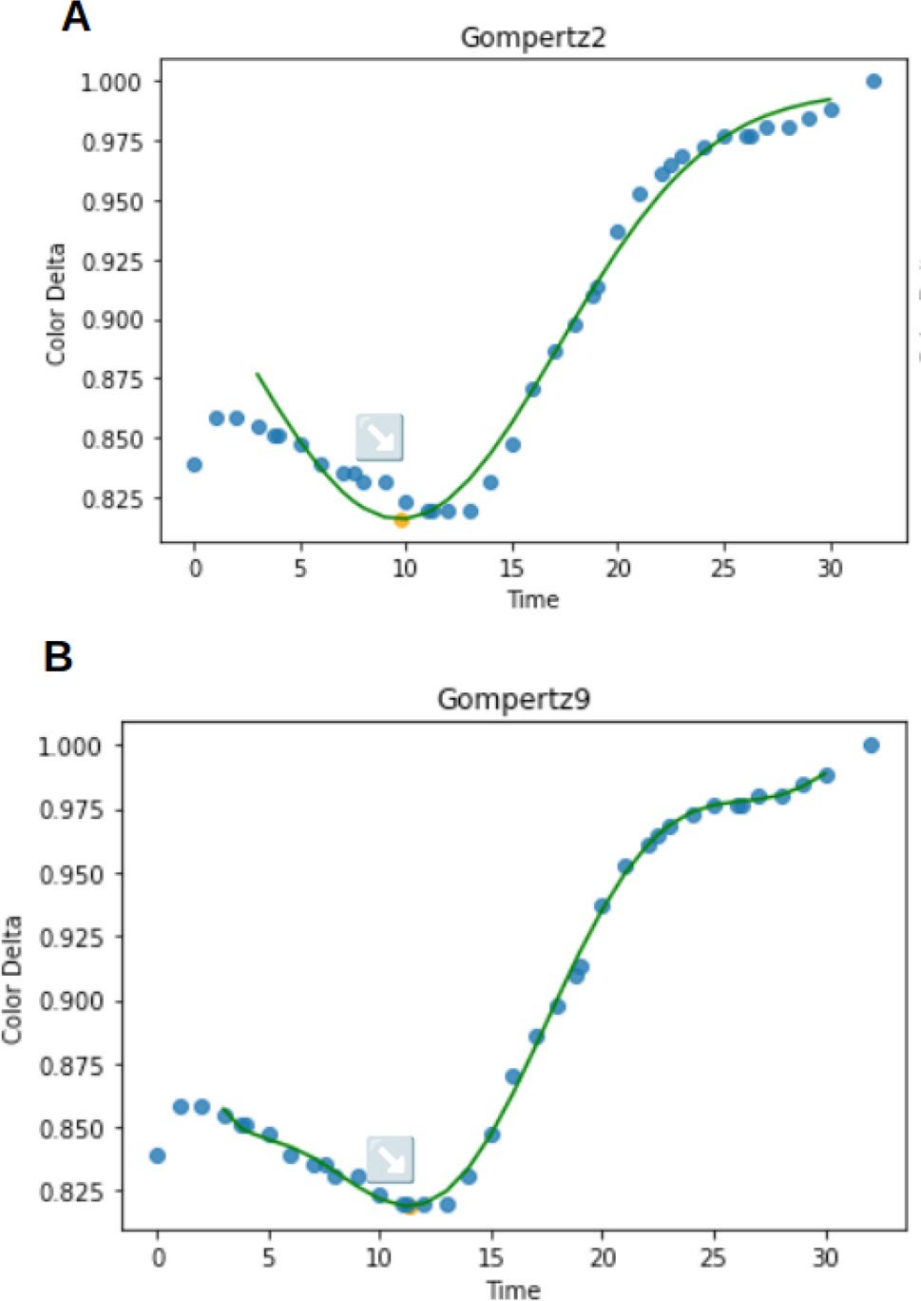
Example of 2 and 9th-degree multimodal Gompertz fit (**A,B**, respectively). Blue points are raw data, the green line is the fitted curve, and the yellow dot (indicated with an arrow) is the second derivative minimum.

The effect of these models when analyzing the Serial Dilution Dataset was also investigated. To do so, the coefficient of determination (R^2^) was recalculated, assuming the relationship between the called parameters (TTP) and the initial log concentration of the target template to be linear (Fig. S5). The 9th-degree multimodal Gompertz model exhibited the highest R^2^, at 77% (Fig. S6). Gompertz2 performed similarly, but being a two-inflection curve, it can only capture a macro pattern of signal decay followed by a rise (Fig. 3). A lower number of parameters may be beneficial to R^2^ values (of the linear regression of TTP against the log of initial target copy number), much of the curve information is lost or corrupted, and the fit is noticeably worse. This is similar to the 4p model compared to a 5p model (Eqs. 2 and 3), where added complexity led to a worse linear regression fit. It is probable that a simple polynomial regression directly using the raw data, followed by second derivative minima calling, could achieve similar results with even fewer parameters.

For this round of analysis, we chose the Quartile coefficient of Distribution (QCD), since we did not remove outliers from the dataset. We also analyzed the resolution of the dilution curve, in the form of fold detectable^38^ values by the calibration curve (Fig. S7). These results corroborate the pattern seen for R^2^, while regarding QCD values, Gompertz9 is comparable to Richard’s four and five-parameter curves. Resolution values exhibit a similar pattern to R^2^, as expected. Lower-order Gompertz models exhibit higher resolution.

**Figure 4:**
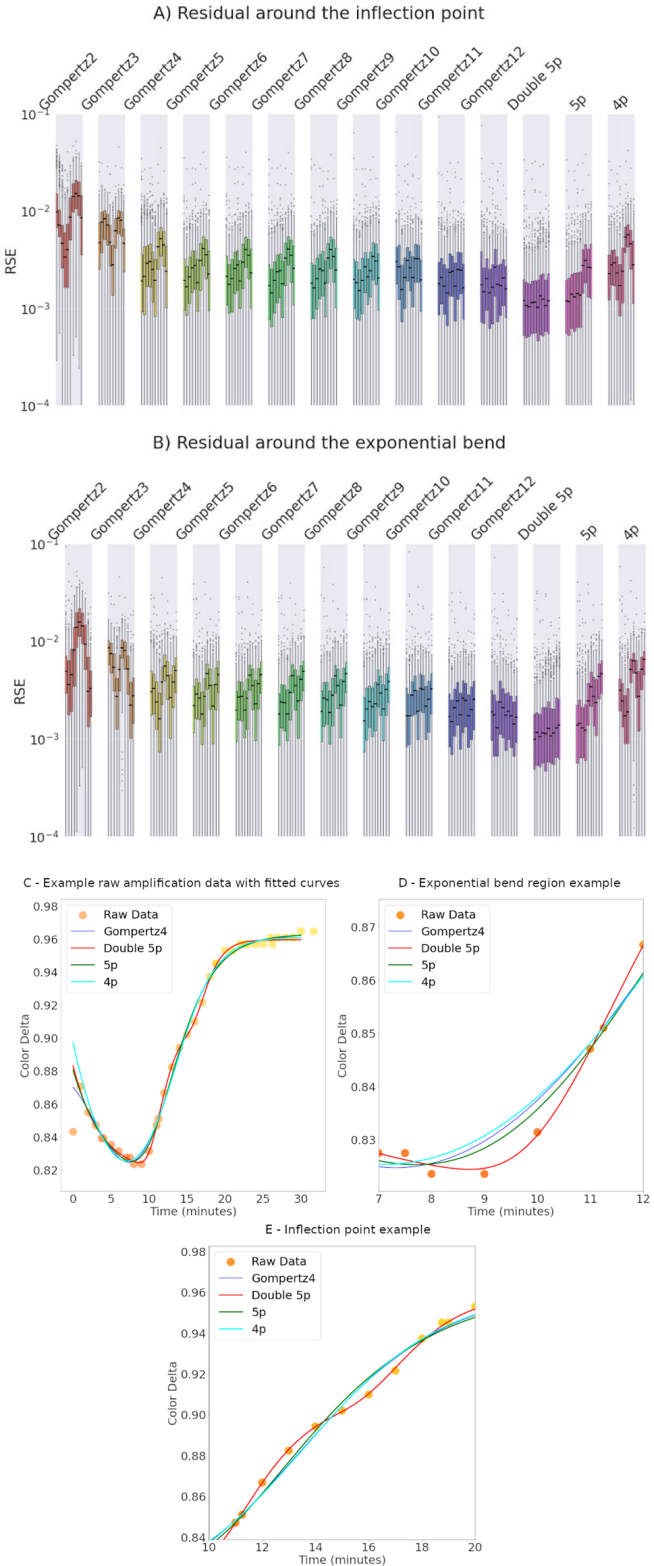
The double 5p sigmoid curve describes key points in the curve. RSE (root square error) is represented in a box plot, with ten points around each key feature: (**A**) inflection point and (**B**) exponential bend. (**C**) Example of raw amplification data with fitted curves [4th-Gompertz, double 5p sigmoid, five and four-parameters models (5p and 4p, respectively)]. (**D**) Example of raw amplification data with fitted curves with a focus on the exponential region of the amplification. (**E**) Example of raw amplification data with fitted curves with a focus on the inflection point of the amplification curve.

To evaluate the performance of the regression models regarding the fit to regions of inflection and exponential growth (C_bend_), we have plotted the Root Square Error (RSE) in 10 points around the inflection point and the C_bend_ for the relevant models (Fig. 4). In terms of fit, the five-parameter model performed similarly to the four-parameter model. Gompertz multimodal models exhibit progressively better fits up to order 5, with diminishing returns for higher orders. The Double Gompertz model displayed the best fits, particularly in the C_bend_ region of the curve. Importantly, this model also avoids truncation patterns where the fitted line crosses the raw data line, instead closely following the curve's profile.

## Discussion

The analysis of colorimetric time course data has become increasingly relevant with the rise of isothermal nucleic acid amplification reactions, especially in the subject of clinical diagnostics. Such reactions are analogous to qPCR amplification curves, but happen continuously and are exposed to much more variation. To validate the use of data processing techniques for results acquired from remote diagnostic assays, we have tested several strategies and used statistical parameters to choose the best-fitting option among these treatments.

We have tested two approaches against the classical sigmoid curves, a multimodal Gompertz^32^ growth curve and a Double 5p sigmoid curve with an exponential baseline. A Gompertz multimodal regression may have any number of inflection points, being dependent on the polynomial expression order inside the exponential expression. The final order is usually chosen to use statistical parameters such as AIC and BIC^32^. These expressions do not assume a saturating baseline behavior and may be more resistant to noisy and unusual data. For both statistics, a 4th order Gompertz growth seems to exhibit the best balance between fit and complexity. That is, Eq. (5) with ${Q}_{\beta }(t)$ equal to a 4th degree polynomial in the form of:

7 $$ \beta_{{4}} *{\text{t}}^{{4}} + \beta_{{3}} *{\text{t}}^{{3}} + \beta_{{2}} *{\text{t}}^{{2}} + \beta_{{1}} *{\text{t}} $$

The AICw for each curve in the Positive Control Dataset was calculated. AICw values seek to indicate if a given model is the best description of the fitted data, and is expressed as a probability. The distribution of these weights was plotted for analysis. Again, a 4th-order Gompertz was the most probable model with a higher frequency than other models, including the classic sigmoidal ones. Interestingly, despite the Double 5p having 13 parameters, its AIC and BIC values are as good as 4th order Gompertz (8 parameters).

It is possible to observe that lower-order models end up being influenced by distant curve features, and thus can produce good correlations to the initial copy number of the target. This happens because the maximum color delta at the end of the reaction also correlates to the initial copy number.

Gompertz9 and the Double 5p, on the other hand, can maintain a good correlation by precisely describing the exponential section of the curve, isolating noise and distortion. This is especially interesting for applications such as machine learning classification^39^, since it provides a wide array of accurate parameters for clustering. Covariance for these models (in the form of QCD) was best for Gompertz9 and Richard’s model variations, once again showing the value of an accurate curve description instead of low complexity for regression models. Among those, however, only the Double 5p model accurately describes the curve in the central region and exponential bend, which are most strongly correlated to the initial target copy number.

## Conclusion

In terms of goodness of fit, a double sigmoid model, combined with an exponential term to account for baseline correction, was the most adequate, describing key regions of the curve with no visually observable overfitting. Although the model includes 13 parameters, its AIC scores are comparable to simpler models, and AICw frequency is consistently more probable for the double sigmoid model than for the simple four-parameter logistic model.

For the Hilab Molecular device tested, we have determined the double Richard’s model as the most adequate to capture central curve geometry with higher accuracy. A downside of this regression model is the fact that it does not adequately capture the continually increasing upper plateaus, and numerous parameters of this model may make convergence difficult. Differential evolution is appropriate for these scenarios, as well as careful bounds value setting. A multimodal Gompertz model, constructed using a 4th-degree polynomial was also found to accurately describe the datasets with a low amount of parameters compared to the previously analyzed Richards models, which can be relevant in some applications.

This work is a step towards standardization of data treatment in high sampling real-time colorimetric LAMP reaction analysis. These findings may be used for assay development, optimization, and also aid in the interpretation of results, allowing a more unbiased parameter calling and easier comparison between assays, as well as quality control monitoring. This could also potentially lead to advances in the development of machine learning categorization of RT-LAMP assays for improved and quicker diagnostics, especially when high volumes of data must be processed.

### Supplementary Information

Supplementary Information 1.

Supplementary Information 2.

## Data Availability

All data used in this study is available in the Supplementary Files.
