# Supplementary material for: Data treatment methods for real-time colorimetric loop-mediated isothermal amplification reactions
Source: Sci Rep. 2023 Sep 1;13:14397. doi: 10.1038/s41598-023-40737-x (PMC10474118; doi:10.1038/s41598-023-40737-x)
Supplement: Supplementary file 2 — Supplementary Information 2. [file 41598_2023_40737_MOESM2_ESM.docx]

**SUPPLEMENTARY INFORMATION**

**Table S1 - Primers used in this study**

| **Primer ID** | **Sequence (5' → 3')** | **Target gene** |
| --- | --- | --- |
| E1_1_F3 | TGAGTACGAACTTATGTACTCAT | E1 Gene |
| E1_1_B3 | TTCAGATTTTTAACACGAGAGT |  |
| E1_1_FIP | ACCACGAAAGCAAGAAAAAGAAGTTCGTTTCGGAAGAGACAG |  |
| E1_1_BIP | TTGCTAGTTACACTAGCCATCCTTAGGTTTTACAAGACTCACGT |  |
| E1_1_LF | CGCTATTAACTATTAACG |  |
| E1_1_LB | GCGCTTCGATTGTGTGCGT |  |
| N2_2_F3 | ACCAGGAACTAATCAGACAAG | N2 Gene |
| N2_2_B3 | GACTTGATCTTTGAAATTTGGATCT |  |
| N2_2_FIP | TTCCGAAGAACGCTGAAGCGGAACTGATTACAAACATTGGCC |  |
| N2_2_BIP | CGCATTGGCATGGAAGTCACAATTTGATGGCACCTGTGTA |  |
| N2_2_LF | GGGGGCAAATTGTGCAATTTG |  |
| N2_2_LB | CTTCGGGAACGTGGTTGACC |  |

**Table S2 - Sequence of control plasmids used for reactions**

| **Target gene*** | **Sequence (5’→ 3’)** |
| --- | --- |
| E1 | ATGTACTCATTCGTTTCGGAAGAGACAGGTACGTTAATAGTTAATAGCGTACTTCTTTTTCTTGCTTTCGTGGTATTCTTGCTAGTTACACTAGCCATCCTTACTGCGCTTCGATTGTGTGCGTACTGCTGCAATATTGTTAACGTGAGTCTTGTAAAACCTTCTTTTTACGTTTACTCTCGTGTTAAAAATCTGAATTCTTCTAGAGTTCCTGATCTTCTGGTCTAA |
| N2 | AACCCAAGGAAATTTTGGGGACCAGGAACTAATCAGACAAGGAACTGATTACAAACATTGGCCGCAAATTGCACAATTTGCCCCCAGCGCTTCAGCGTTCTTCGGAATGTCGCGCATTGGCATGGAAGTCACACCTTCGGGAACGTGGTTGACCTACACAGGTGCCATCAAATTGGATGACAAAGATCCAAATTTCAAAGATCAAGTCATTTTGCTGAATAAGCATAT |

*Target gene sequences were cloned in the EcoRV restriction site of a pUC57 plasmid backbone.


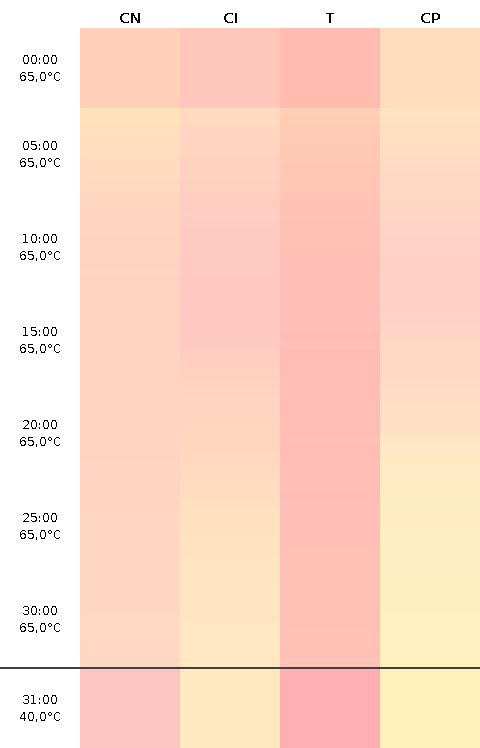

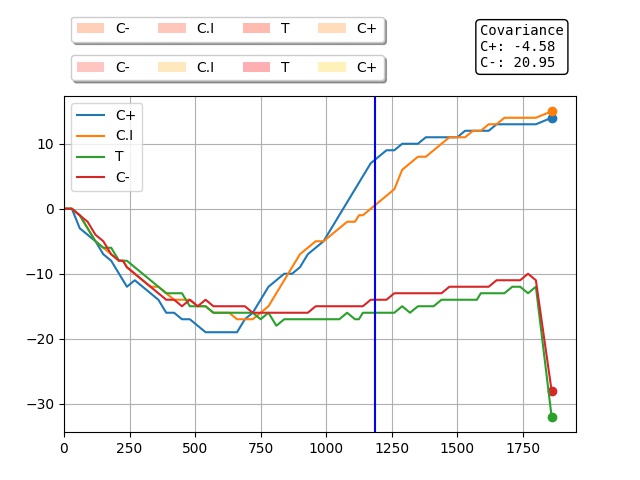


**Figure S1** - An example of color series data (A) and its graphic representation (B) obtained from the Hilab Molecular PoC device (in the x-axis is the time, in seconds).


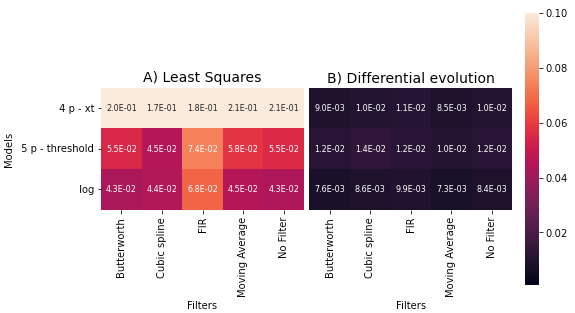


**Figure S2** - Heatmap of the mean squared error of regressions using least squares (A) and differential evolution (B) methods, and different logistic models. In the vertical are the different filters used, and in the horizontal is the different curve models (4p = four-parameters model; 5p = five-parameters; log = five-parameters log model). The more intense is the color, lower is the mean squared error.


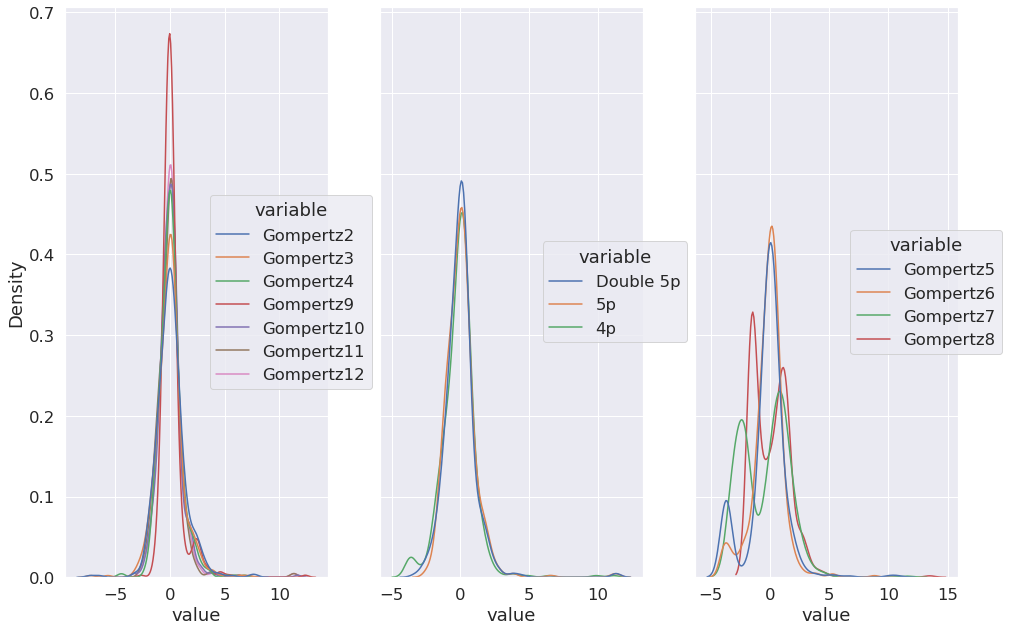
**Figure S3** - **KDE of AICw distributions for different models.** Called parameters for each model were used to build KDE plots, in order to analyze variability between different models on the same dataset. Data were plotted in three groups from left to right: best multimodal Gompertz, Richard’s models, and worst Gompertz models. The best Gompertz model is comparable to Richard’s models. Worse Gompertz models are unstable. Gompertz9 outperformed all the models tested.


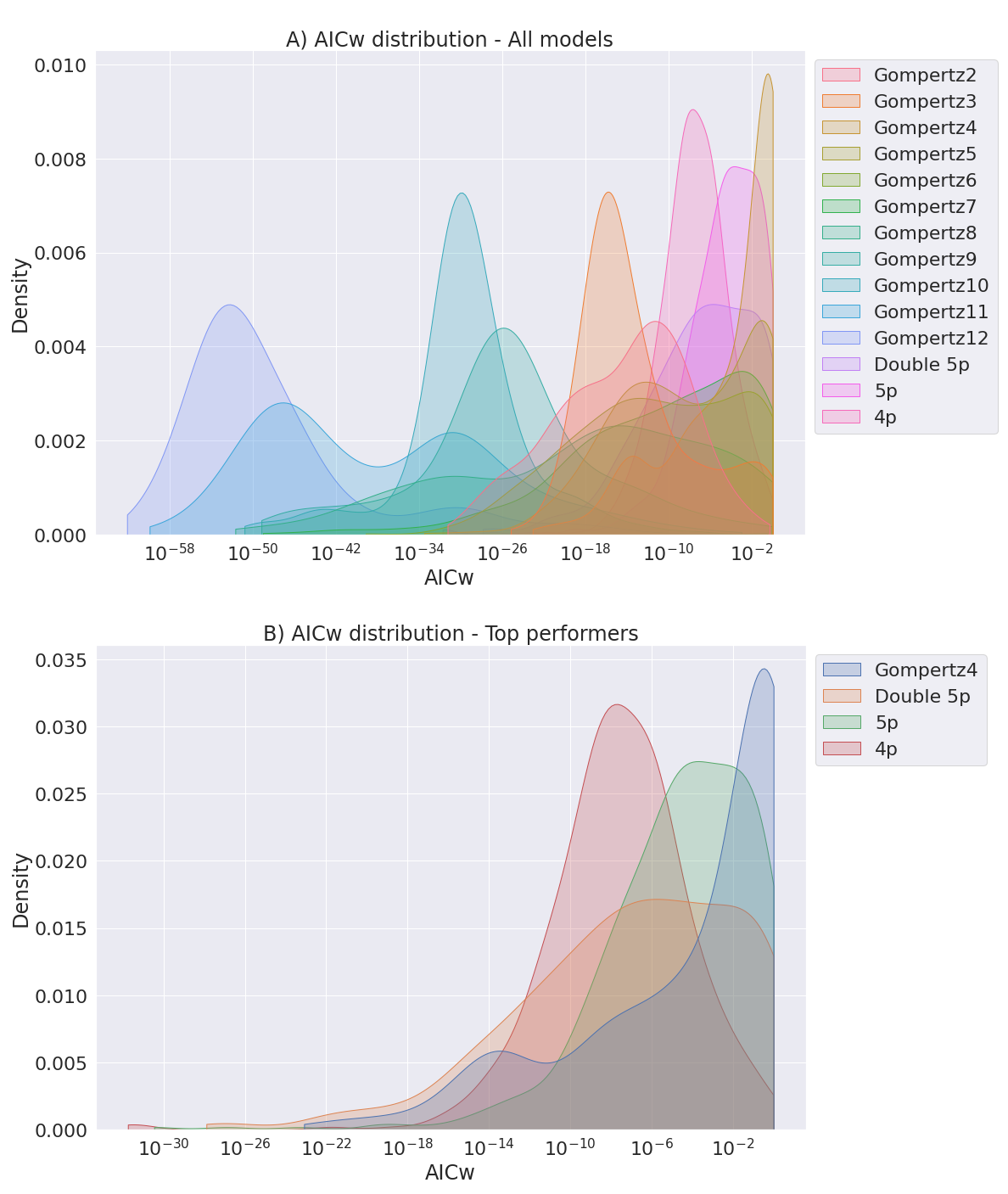


**Figure S4** **- Distribution of AICw comparing different models.** Individual AIC weights distribution, comparing 2 to 12-order polynomial Gompertz, Double 5-parameter sigmoid, regular five-parameter (5p), and four-parameter (4p) sigmoids. In the Y-axis is the density and the X-axis is the AICw values. **A)** All models plotted. **B)** Top performing models (4-order polynomial Gompertz, double 5-parameter sigmoid, regular five and four-parameter sigmoids).


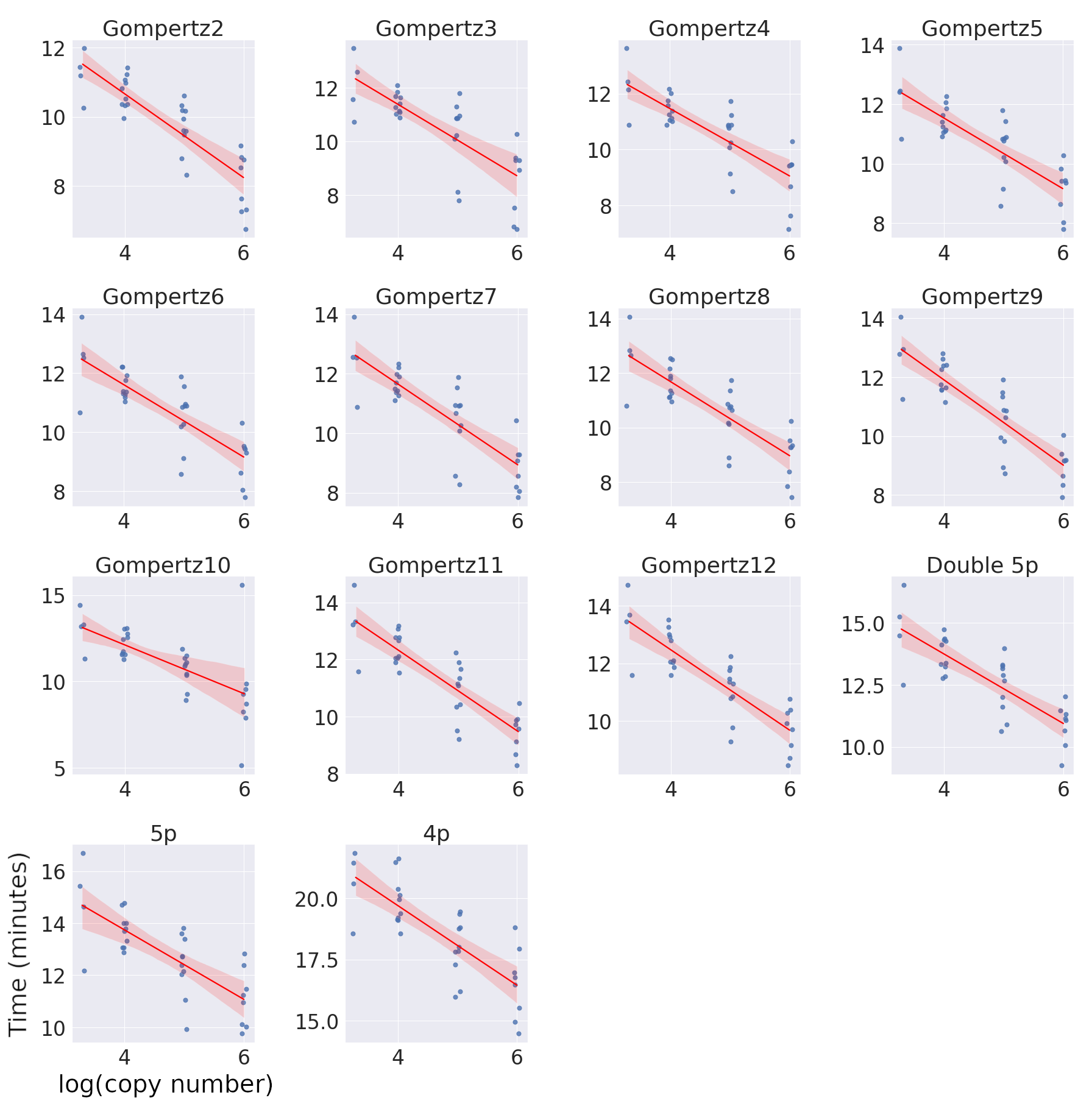


**Figure S5** - Linear regression of the log of copy number (X-axis) by Time to Positivity (Y-axis), for different models (2 to 12-order polynomial Gompertz, Double 5-parameter sigmoid, regular five-parameter (5p), and four-parameter (4p) sigmoids). The red translucent bands represent 95% Confidence Interval (CI) and the blue dots represent the replicates. Each graph contains a serial dilution of log3 to 6 from the synthetic positive control (SARS-CoV-2).


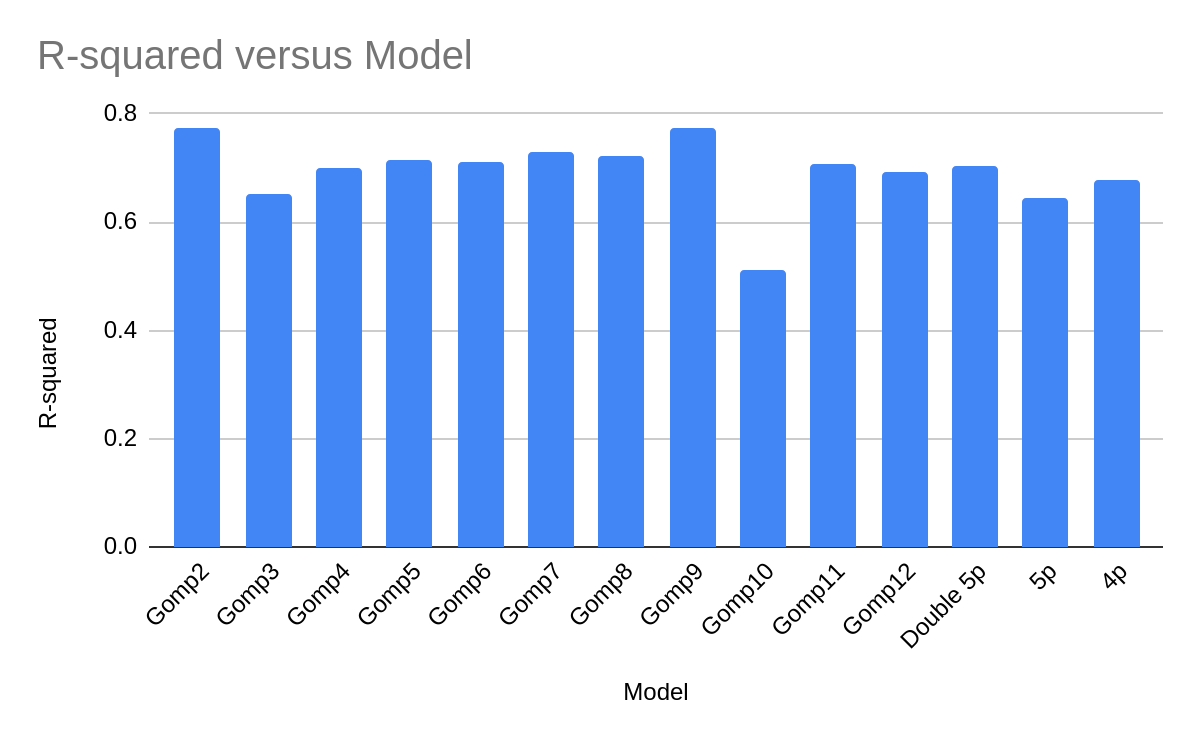
**Figure S6** - **A 9**^th^**-degree multimodal Gompertz yields the highest coefficient of determination (R²)**. The coefficient of determination calculated from the linear regression of the copy number (log) against TTP in the Y-axis and in the X-axis the different curve models tested (2 to 12-order polynomial Gompertz, Double 5-parameter sigmoid, regular five-parameter (5p), and four-parameter (4p) sigmoids).


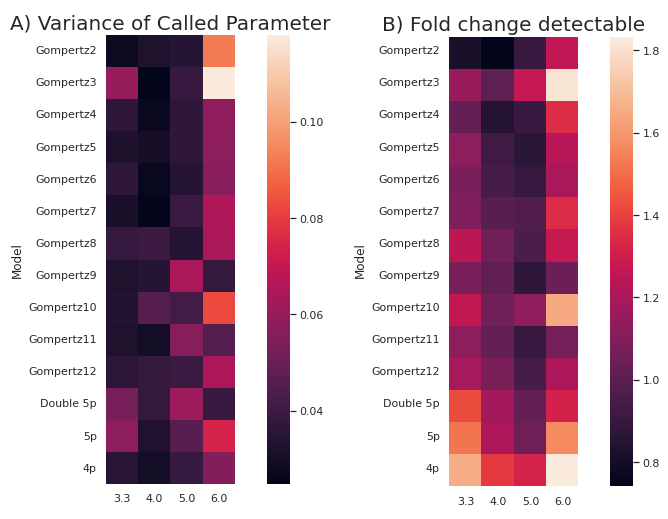


**Figure S7** - **A 9**^th^**-degree multimodal Gompertz performs similarly to the models based on Richard’s curve.** The x-axis represents the log of the synthetic positive control copy number and the y-axis represents the different curve models. A) A heatmap displaying Quartile coefficient of Distribution (QCD) values (lower values, e.g. the darkest squares, represent better performance). B) A heatmap representing the resolution of the calibration curve in the form of detectable fold increase (lower values, e.g. the darkest squares represent better performance). More intense colors represent lower values.
